# The Atypical MAP Kinase MAPK15 Is Required for Lung Adenocarcinoma Metastasis via Its Interaction with NF-κB p50 Subunit and Transcriptional Regulation of Prostaglandin E2 Receptor EP3 Subtype

**DOI:** 10.3390/cancers15051398

**Published:** 2023-02-22

**Authors:** Fei-Yuan Yu, Qian Xu, Xiao-Yun Zhao, Hai-Ying Mo, Qiu-Hua Zhong, Li Luo, Andy T. Y. Lau, Yan-Ming Xu

**Affiliations:** 1 Laboratory of Cancer Biology and Epigenetics, Department of Cell Biology and Genetics, Shantou University Medical College, Shantou 515041, China; 2 Laboratory of Molecular Pathology, Department of Pathology, Shantou University Medical College, Shantou 515041, China

**Keywords:** MAPK15, EP3, p50, LUAD, metastasis

## Abstract

**Simple Summary:**

Due to the lack of effective early diagnostic markers for lung cancer and the rich blood circulation in the lungs, it is very easy to cause lymph node metastasis and distant metastasis of lung cancer, making lung cancer as one of the top ten cancer types with the highest mortality rate in the world. This study found that MAPK15 is highly expressed in the tissues of patients with lung adenocarcinoma lymph node metastasis, and MAPK15 interacts with p50 to regulate the expression of EP3 at the transcriptional level, thereby promoting cancer cell migration. This suggests that MAPK15 plays a key role in the metastasis of lung cancer cells, and MAPK15 can be used as a molecular marker for the early diagnosis or prognosis assessment of lung cancer. Its molecular mechanism for regulating lung cancer metastasis can provide valuable information and insights on novel therapeutic options at molecular levels.

**Abstract:**

Studying the relatively underexplored atypical MAP Kinase MAPK15 on cancer progression/patient outcomes and its potential transcriptional regulation of downstream genes would be highly valuable for the diagnosis, prognosis, and potential oncotherapy of malignant tumors such as lung adenocarcinoma (LUAD). Here, the expression of MAPK15 in LUAD was detected by immunohistochemistry and its correlation with clinical parameters such as lymph node metastasis and clinical stage was analyzed. The correlation between the prostaglandin E2 receptor EP3 subtype (EP3) and MAPK15 expression in LUAD tissues was examined, and the transcriptional regulation of EP3 and cell migration by MAPK15 in LUAD cell lines were studied using the luciferase reporter assay, immunoblot analysis, qRT-PCR, and transwell assay. We found that MAPK15 is highly expressed in LUAD with lymph node metastasis. In addition, EP3 is positively correlated with the expression of MAPK15 in LUAD tissues, and we confirmed that MAPK15 transcriptionally regulates the expression of EP3. Upon the knockdown of MAPK15, the expression of EP3 was down-regulated and the cell migration ability was decreased in vitro; similarly, the mesenteric metastasis ability of the MAPK15 knockdown cells was inhibited in in vivo animal experiments. Mechanistically, we demonstrate for the first time that MAPK15 interacts with NF-κB p50 and enters the nucleus, and NF-κB p50 binds to the EP3 promoter and transcriptionally regulates the expression of EP3. Taken together, we show that a novel atypical MAPK and NF-κB subunit interaction promotes LUAD cell migration through transcriptional regulation of EP3, and higher MAPK15 level is associated with lymph node metastasis in patients with LUAD.

## 1. Introduction

The incidence of lung cancer is high among malignant tumors, which seriously affects human health. The mortality rate of lung cancer patients is high because lung cancer is usually in an advanced stage when diagnosed, with lymph node metastasis or even distant metastasis. Radiotherapy and chemotherapy have very limited therapeutic effects on advanced lung cancer. Targeted therapies, such as the use of targeted drugs EGFR tyrosine kinase inhibitors [1,2], can improve the survival of lung cancer patients to a certain extent, but they still face the problem of chemotherapy resistance, recurrence of targeted therapy, etc. Therefore, it is still not possible to effectively control the malignant development of lung cancer [2]. The study of molecular markers related to lung cancer metastasis and their corresponding molecular mechanisms still needs to be further explored.

The classical mitogen-activated protein kinases (MAPKs, e.g., ERK1/2, p38, and JNK/SAPK) play important roles in regulating gene expression, cell growth, proliferation, etc. Atypical MAPKs such as ERK3, ERK4, and NLK (nemo-like kinase) also play critical roles in many cellular responses [3,4]. MAPK15, alias extracellular signal-regulated kinase 7/8 (ERK7/8), is the most recently discovered atypical MAPK. Current research indicates that MAPK15 can promote the transformation of colon cancer by mediating the activation of the transcription factor c-Jun [5,6] or promoting the growth of gastric cancer cells [7]. MAPK15 has also been found to interact with autophagy-related proteins such as GABARAP and LC3 to control tumor development [8]. In addition, MAPK15 can be activated by carcinogenic factors such as RET/PTC3 [9] or involved in the regulation of telomerase activity [10] to participate in the development of tumors. Recently, our group has reported that MAPK15 can promote arsenic trioxide-induced apoptosis, as well as boosting the efficacy of combination therapy with cisplatin and TNF-α, in lung cancer cells [11,12]. At present, research about the function of MAPK15 is still limited, and its role in lung cancer metastasis remains unclear.

EP3 is one of the four G protein-coupled receptors of prostaglandin E2 (PGE2), which plays an important role in cell proliferation, differentiation, apoptosis, cardiovascular system regulation, and inflammation. It has been reported that tumor angiogenesis and tumor cell growth were significantly inhibited in a mouse lung cancer model with EP3 knocked out [13]. Yamaki et al. found that PGE2 promotes the growth of lung adenocarcinoma (LUAD) cell line A549 via the EP3 receptor-activated Src signaling pathway [14]. However, the molecular mechanism of EP3 in regulating lung cancer progression is still not fully clarified.

In this study, we detected the expression of MAPK15 in lung cancer tissues, and found that the expression of MAPK15 is positively correlated with lymph node metastasis in LUAD patients; remarkably, our results showed that the expression of EP3 was transcriptionally regulated by MAPK15, and the expression of EP3 was positively correlated with the expression of MAPK15 in LUAD tissues. Furthermore, we revealed the first time that MAPK15 promotes the expression of EP3 by interacting with p50, thereby enhancing the migration of lung cancer cells.

## 2. Materials and Methods

### 2.1. Immunohistochemistry

Lung cancer tissue microarray (BC041115c, US Biomax, Rockville, MD, USA) was purchased and all human tissues were collected according to HIPPA-approved protocols as described by US Biomax (https://www.biomax.us/FAQs, accessed on 14 June 2022). Immunohistochemistry was performed to detect the expression of MAPK15 and EP3. Briefly, tissue microarray was deparaffinized thrice in xylene (10 min for each) and rehydrated in gradient series ethanol (100%, 95%, 90%, 90%, 5 min for each), respectively. After being rinsed with water, tissue slides were incubated with 3% hydrogen peroxide for 40 min to block endogenous peroxidase. Tissue slides were then rinsed with PBS and immersed in 0.01 M citrate acid antigen retrieval solution and heated at 98 °C for 20 min using a water bath. After natural cooling, tissue slides were washed with PBS and incubated with 5% BSA for 30 min. Tissue slides were then incubated with MAPK15 [15] or EP3 (Cat. 101760, Cayman Chemical, Ann Arbor, USA) antibody at 4 °C overnight. After being rinsed with PBS, tissue slides were incubated with secondary antibody for 45 min at RT. Subsequently, tissue slides were washed with PBS and reacted with 3,3′-diaminobenzidine (DAB, Zhongshan Golden Bridge Inc. Beijing, China) and counterstained with hematoxylin. Then, tissue slides were mounted with glycerogelatin and photographed with a light microscope.

Immunostaining of tissue microarray were scored according to immunoreactive score (IRS) [16,17]. Each tissue in the microarray was semiquantitatively scored for intensity (0, absent; 1, weak; 2, moderate; 3, strong) and extent of staining (percentage of the positive tumor cells: 0, ≤5%; 1, 6–25%; 2, 26–50%; 3, 51–75%; 4, >75%). Intensity and extent of each tissue were multiplied to give a composite score: 0–3, deemed as low expression, “−”); 4–12, deemed as high expression (4–6, “+”; 7–9, “++”; 10–12, “+++”).

### 2.2. Cell Culture and Transfection

All cells were grown at 37 °C in a 5% CO_2_ incubator. HEK293T, H1299, and A549 cells were purchased from ATCC Cell Bank of the Chinese Academy of Sciences (Shanghai, China) and maintained in MEM, RPMI-1640, or F12-K medium supplemented with 10% FBS and 1% PS, respectively. MAPK15 stable knockdown LUAD H1299 cells (H1299-shMAPK15) and control cells (H1299-shCtrl) were established previously [12]. For transfection, cells were mixed with siRNA/plasmids-polyethylenimine mixture and cultured for the indicated time point. Negative control siRNA (siN05815122147) and siRNA duplexes against EP3 were purchased from IGE Biotechnology LTD (Guangzhou, China) and listed in Supplementary Table S1.

### 2.3. RNA Extraction, cDNA Synthesis, and Real-Time PCR

RNA was extracted using RNAiso Plus (Takara, Dalian, China) from cells. Then, cDNA was synthesized using GoScript™ Reverse Transcription Mix (Promega, Madison, WI, USA) by following the manufacturer’s instructions. Specific primers were used, and real-time PCR was performed using GoTaq qPCR Master Mix (Promega, Madison, WI, USA) on Applied Biosystems 7500 Real-Time PCR System. The 2^ΔΔCT^ method was used to calculate the relative expression of target genes compared to internal control (β-Actin) as described previously [18]. Primers were synthesized by IGE Biotechnology LTD (Guangzhou, China) and listed in supplementary Table S1.

### 2.4. Immunoblot Analysis

Equivalent amounts of extracted protein were resolved by 10% SDS-PAGE and transferred onto polyvinylidene fluoride membranes. The membranes were blocked with 5% nonfat milk in PBS containing 0.05% Tween 20 followed by incubation with primary antibody overnight at 4 °C. After reacting with primary antibody, membranes were incubated with secondary antibody and proteins were visualized with ECL reagent using Tanon 5200 system (Tanon, Shanghai, China). The optical density of each protein band was quantified by Gel-Pro Analyzer 4 (Toyobo, Osaka, Japan) software. Original blots and blot quantification are shown in Figures S3–S5, S7 and S8.

### 2.5. Transwell Assay

Transwell assay was performed as described previously [16]. Briefly, 3.0 × 10^4^ cells were seeded in the upper compartment of transwell inserts with 8 μm microporous membrane (cat no. 3422, Corning Inc., Corning, NY, USA). After being incubated for 24 h, unmigrated cells on the upper surface of the microporous membrane were wiped using a cotton swab. Cells on the lower surface of the microporous membrane were fixed with 4% PFA for 20 min and subsequently stained with 0.1% crystal violet for 15 min. The transwell chamber was rinsed with PBS to remove excess crystal violet, and images of migrated cells were captured using an Axiovert 40 CFL microscope (Carl Zeiss AG, Oberkochen, Germany) with CCD camera (magnified 100×). Finally, the crystal violet in the migrated cells was dissolved with 33% acetic acid, and absorbance was measured at OD_595_.

### 2.6. Immunofluorescence and Confocal Microscopy

Cells seeded on coverslip in 6-well plate were incubated for the indicated time point and fixed with 4% PFA for 15 min. After being rinsed with PBS, cells were permeabilized for 10 min with PBS containing 0.25% Triton X-100. Subsequently, cells were incubated with 5% BSA for 30 min to block unspecific binding of antibodies. Then, cells were incubated with primary antibody in a humidified chamber at 4 °C overnight. After decanting of primary antibody solution, cells were washed with PBS and incubated with secondary antibody for 1.5 h at room temperature in the dark. Coverslips were counterstained with 1 μg/mL Hoechst 33342 and mounted with mounting medium. Images were captured with Axiovert 40 CFL Microscope (Carl Zeiss AG, Germany) or Zeiss lsm 800 confocal microscope (Carl Zeiss AG, Germany).

### 2.7. In Vivo Peritoneal Metastasis Assay

In vivo peritoneal metastasis assay was performed as described previously [19]. Briefly, 5 × 10^6^ MAPK15 stable knockdown H1299 or control cells in 200 μL of phosphate-buffered saline were injected intraperitoneally into BALB/c nude mice (Beijing Vital River Animal Technology Co., Ltd., Beijing, China, licensed by Charles River). After 7 weeks, the mice were sacrificed, and tumor nodules were quantified.

### 2.8. Co-Immunoprecipitation

HEK293T cells cultured in 10 cm dish were transfected with pcDNA4/Xpress-MAPK15 plasmids [15] and incubated for 24 h. Prior to immunoprecipitation, 1 μg of Xpress antibody or normal IgG was pre-adsorbed with 20 μL Protein A/G Sepharose slurry for 2 h at 4 °C with rotation. After transfection, cells were harvested and lysed with NP-40 lysis buffer using repetitive freeze-thawing method. An amount of 300 μg of lysates to be used for immunoprecipitation was precleared with 20 μL Protein A/G Sepharose at 4 °C for 1 h with rotation. The supernatant was then incubated with the Xpress antibody–Protein A/G Sepharose complexes overnight at 4 °C with rotation (anti-mouse IgG was used as negative control). In total, 10% of the supernatant was used as input. The Sepharose beads were collected by centrifugation and washed extensively in 500 μL of lysis buffer, and eluted in 20 μL of SDS sample buffer by heating to 98 °C for 5 min. After centrifugation at 10,000× *g*, the supernatant was collected for immunoblot analysis.

### 2.9. Chromatin Immunoprecipitation Assay

Chromatin immunoprecipitation assay was performed using SimpleChIP^®^ Enzymatic Chromatin IP Kit (Cell Signaling Technology, Danvers, MA, USA). Briefly, formaldehyde cross-linked H1299 cells were lysed, and chromatin was digested with micrococcal nuclease into DNA/protein fragments. Then, p50 antibody (Santa Cruz Biotechnology, Dallas, TX, USA) was added and the complex is captured by protein G magnetic beads. Seven p50 binding sites (site1–site7) in the EP3 promoter region (−2000 bp) were predicted by JASPAR databases and PCR was used to detect p50 binding.

### 2.10. Vector Construction and Luciferase Reporter Assay

Five repeats of p50 binding sequence (site5, sequence: GGGGCTTCCC) and 12 bp linker sequences with AflII and NsiI sites were synthesized by IGE Biotechnology LTD (Guangzhou, China) and ligated to a modified pJC6-GL3 plasmid [11] to construct luciferase reporter plasmid (5 × p50-Luc). Then, the 5 × p50-Luc plasmid was co-transfected with/without pCMV-p50 plasmid into equal amount of H1299 cells in 12-well plate. Afterward, cells were lysed for luciferase assay following manufacturer’s instructions (dual-luciferase reporter assay system, Promega, Madison, WI, USA).

### 2.11. Statistical Analysis

Mean comparisons were performed using the GraphPad Prism 8 for unpaired *t*-test. Fisher’s exact test was used to study the correlation between MAPK15 expression and clinical parameters. Spearman rank correlation analysis was used to compare the correlation between the expression of MAPK15 and EP3 in lung cancer tissues using SPSS 19 software. The above statistical analysis was two-tailed; *p* < 0.05 suggested that the difference was statistically significant.

## 3. Results

### 3.1. MAPK15 Is Correlated with Lymph Node Metastasis in LUAD Patients

**Figure 1:**
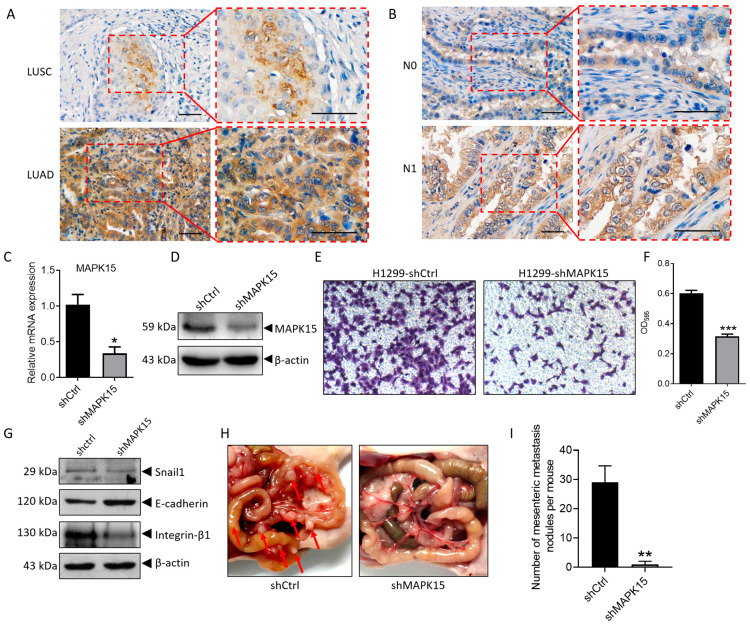
Knockdown of MAPK15 inhibits H1299 cell migration in vitro and in vivo. (**A**) MAPK15 staining in lung squamous cell lung carcinoma and adenocarcinoma tissues. (**B**) MAPK15 staining in LUAD tissues without lymph node involvement (N0) was compared with tissue with lymph node involvement (N1). Scale bar represents 60 μm. (**C**,**D**), real-time PCR and immunoblot analysis were used to detect the mRNA level (**C**) and protein level (**D**) of MAPK15 in H1299 cells. (**E**,**F**), transwell assay was used to detect the migration ability of H1299-shCtrl and H1299-shMAPK15 cells, migrated cells were stained with crystal violet (**E**) and absorbance of solubilized crystal violet was shown as bar chart graph (**F**). (**G**) The expression of Snail1/E-cadherin/Integrin-β1 was detected in H1299-shCtrl and H1299-shMAPK15 cells. (**H**) Metastatic nodules (red arrows) on intestinal mesentery of BALB/c nude mice. (**I**) Number of mesenteric metastasis nodules per mouse. * *p* < 0.05, ** *p* < 0.01, *** *p* < 0.001, Student’s *t*-test.

To study the role of MAPK15 in lung cancer, we analyzed the relationship between MAPK15 and clinical–pathological parameters such as age, gender, depth of tumor invasion, lymph node metastasis, distant metastasis, tumor differentiation, clinical stage, etc. We found that there was a positive correlation between MAPK15 expression and lymph node metastasis (*p* = 0.012) as well as clinical stage (*p* = 0.033) (Supplementary Table S2). The expression of MAPK15 is higher in patients with lymph node metastasis (N1 + N2) as compared to patients without lymph node metastasis (N0) (Supplementary Table S2). Other clinical–pathological parameters such as age, gender, depth of tumor invasion, distant metastasis, and tumor differentiation were not significantly correlated with the expression of MAPK15 (Supplementary Table S2). Adenocarcinoma and squamous cell carcinoma are major types of non-small-cell lung cancer (NSCLC). As compared to squamous cell carcinoma, we revealed that the expression of MAPK15 is relatively higher in adenocarcinoma (Figure 1A, Supplementary Table S3) and is associated with lymph node metastasis (*p* = 0.013) (Table 1, Figure 1B).

### 3.2. Knockdown of MAPK15 Inhibits H1299 Cell Migration In Vitro and Metastasis In Vivo

The above results indicate that MAPK15 is expressed more highly in lymphatic metastatic LUAD. In MAPK15 stable knockdown LUAD H1299 cells (Figure 1C,D), cell migration was significantly inhibited (Figure 1E,F). The expression of Snail1 was decreased in MAPK15 knockdown cells (Figure 1G), which can down-regulate the expression of E-cadherin by post-translational modifications such as deacetylation and methylation during EMT [20]. Consequently, the expression of epithelial marker E-cadherin was increased, while mesenchymal marker integrin β1 is decreased after MAPK15 knockdown (Figure 1G). Then, we performed an in vivo peritoneal metastasis assay using H1299-shMAPK15 cells and found that loss of MAPK15 significantly reduces metastasis to mesentery in vivo (Figure 1H,I). The above results indicate that H1299 cells undergo mesenchymal–epithelial transition after MAPK15 knockdown, thereby decreasing migration and metastasis.

### 3.3. MAPK15 Regulates the Expression of Migration-Related Gene EP3

**Figure 2:**
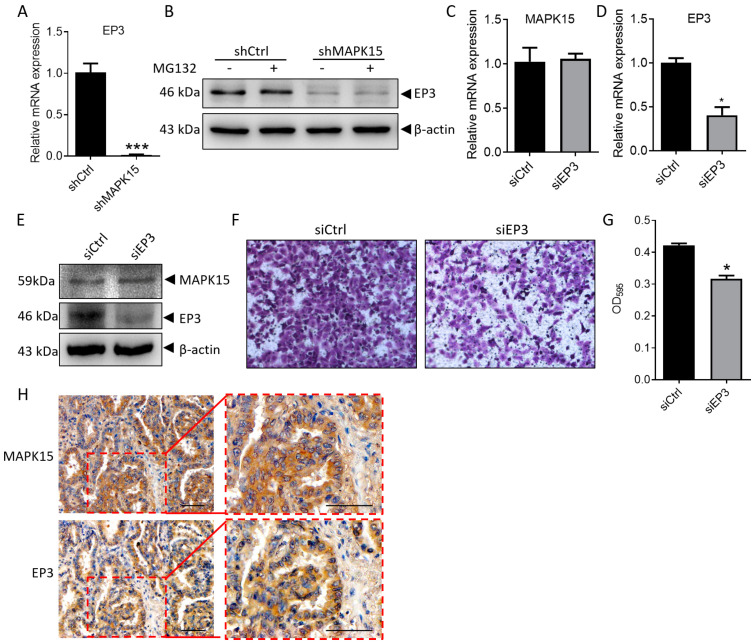
MAPK15 regulates the expression of migration-related gene EP3. (**A**) The expression of EP3 mRNA was detected in H1299-shctrl and H1299-shMAPK15 cells by real-time PCR. (**B**) H1299-shCtrl and H1299-shMAPK15 cells were treated with/without 10 μmol/L MG132 for 4 h, then the expression of EP3 was detected by immunoblot analysis. (**C**–**E**) An amount of 40 μmol/L of negative control siRNA and EP3 siRNA were transfected into H1299 cells, respectively, for 36 h and the expression of MAPK15/EP3 were detected. (**F**,**G**) H1299 cells transfected with negative control siRNA and EP3 siRNA for 36 h were seeded in transwell chamber for 24 h, then migrated cells were stained with crystal violet (**F**) and absorbance of solubilized crystal violet are shown as bar chart graph (**G**). (**H**) Serial section of the same LUAD tissue shows the similar expression pattern of MAPK15 and EP3. Scale bar represents 60 μm. * *p* < 0.05, *** *p* < 0.001, Student *t* test.

It has been reported that the expression of MMP2 was depressed in EP3 knock-out mice under hypoxic stress [21], which indicates a correlation between the expression of MMP2 and EP3. Our results showed that MMP2 was down-regulated in MAPK15 knockdown H1299 cells (Figure S1). To investigate whether EP3 is involved, we detect the expression of EP3 in H1299-shCtrl and H1299-shMAPK15 cells. We found that the mRNA and protein level of EP3 was significantly decreased in MAPK15-deficient cells (Figure 2A,B) and the protein level of EP3 was not affected by proteasome inhibitor MG132 (Figure 2B), suggesting that the decreased EP3 in H1299-shMAPK15 cells was transcriptionally regulated. Moreover, the migration of H1299 cells was inhibited (Figure 2F,G) after EP3 was knocked down (Figure 2C–E). The decreased EP3 in MAPK15 knockdown cells suggested that there might be a correlation between the expression patterns of these two molecules. Then, we detected the expression of EP3 in a serial section from the same tissue that we stained with MAPK15 antibody and found that the expression of EP3 is positively correlated with MAPK15 (r = 0.589, *p* < 0.001, Figure 2H and Table 2). Taken together, the above results show that MAPK15 affects cell migration through the regulation of EP3.

### 3.4. MAPK15 Interacts with NF-κB p50 Subunit and NF-κB p50 Transcriptionally Regulates EP3 Expression by Binding to EP3 Promoter

**Figure 3:**
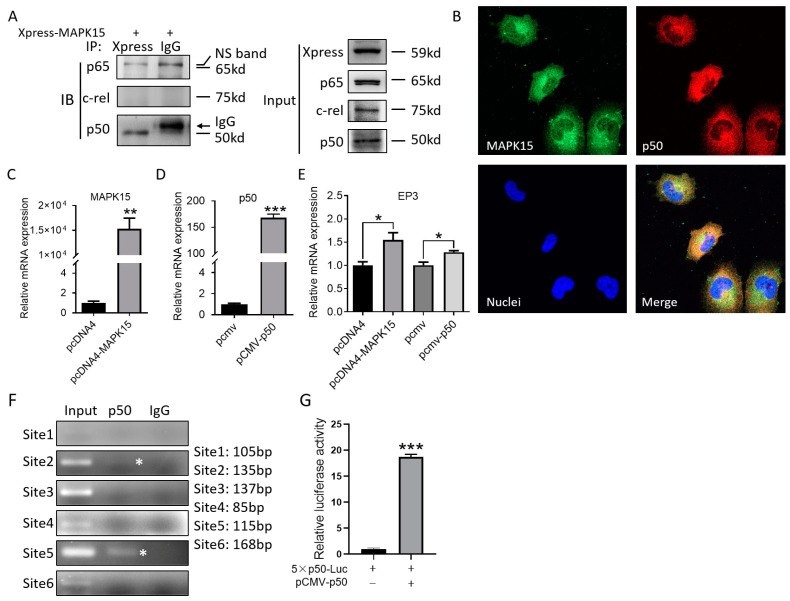
MAPK15 interacts with NF-κB p50 and p50 promotes EP3 expression by binding to EP3 promoter. (**A**) Immunoprecipitated proteins were resolved and the presence of MAPK15 and p50/c-rel/p65 were detected by anti-Xpress or anti-p50/c-rel/p65 antibodies. (**B**) The localization of MAPK15 and p50 in 4% paraformaldehyde-fixed H1299 cells were detected. (**C**–**E**) H1299 cells transfected with 2 μg pcDNA4, pcDNA4/Xpress-MAPK15, pCMV, and pCMV-p50 and the expression of MAPK15 (**C**)/p50 (**D**)/EP3 (**E**) was detected by real-time PCR. (**F**) Chromatin immunoprecipitation assay was used to detect the binding of p50 to EP3 promoter region; the asterisk indicates the immunoprecipitated EP3 promoter region. Original gels and gel quantification are shown in Figure S6. (**G**) In this study, 5 × p50-Luc plasmid was transfected with/without pCMV-p50 in H1299 cells and luciferase reporter assay was performed to detect the luciferase activity. * *p* < 0.05, ** *p* < 0.01, *** *p* < 0.001, Student’s *t*-test.

The molecular mechanism of how EP3 is transcriptionally regulated by MAPK15 is unknown. It has been reported that the expression of MAPK15, NF-κB1 (p50), and NF-κB2 (p52) were obviously decreased in ovarian cancer cell lines [22], which indicate there are correlations between MAPK15 and the NF-κB family. To investigate the relationship between MAPK15 and NF-κB family members, we transfected the pcDNA4/Xpress-MAPK15 plasmid into 293T cells and the immunoprecipitation assay revealed that MAPK15 interacts with p50 but not p65 and c-rel (Figure 3A). We also detected the localization of MAPK15 and p50 in H1299 cells by confocal microscopy and found that MAPK15 is distributed both in the cytoplasm and nucleus, and colocalizes with p50 (Figure 3B), indicating that there is an interaction between these two proteins in LUAD cells which might contribute to the expression of EP3. To study the relationship between MAPK15/p50 and EP3, we overexpressed MAPK15 (Figure 3C) and p50 (Figure 3D) in H1299 cells and found that the expression of EP3 was increased (Figure 3E), which indicates that MAPK15 and p50 positively regulate the transcription of EP3. The chromatin immunoprecipitation assay found that p50 binds to two p50 binding motifs in the EP3 promoter (Figure 3F, site2 and site5). Subsequently, we chose site 5 (Figure 3F) to construct the luciferase reporter plasmid and co-transfect with/without pCMV-p50 in H1299 cells for the luciferase reporter assay. Our results indicate that the luciferase activity is significantly increased in cells overexpressed with p50 (Figure 3G), which revealed that p50 can transcriptionally regulate EP3 by binding to the EP3 promoter.

### 3.5. TNF-α Promotes H1299 Cell Migration through Induction of MAPK15-NF-κB p50 Nuclear Localization and EP3 Expression

**Figure 4:**
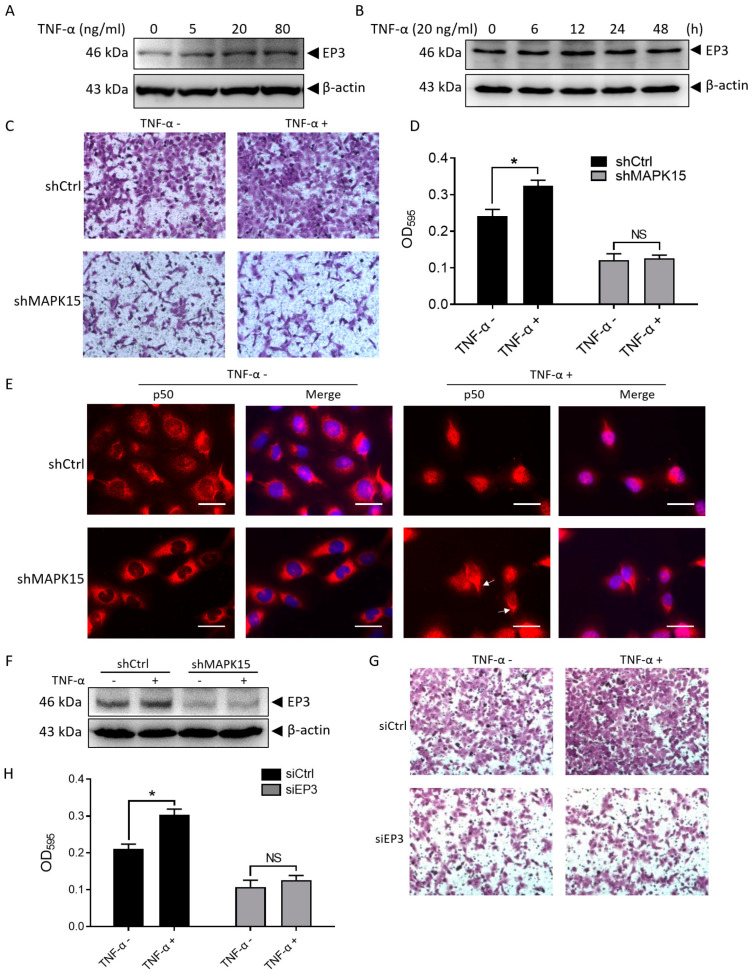
TNF-α promotes H1299 cell migration through induction of EP3 expression. (**A**) EP3 was detected in H1299 cells treated with different concentrations of TNF-α. (**B**) EP3 was detected in H1299 cells treated with 20 ng/mL TNF-α for different time points. (**C**,**D**) Transwell assay was used to detect migration effect of H1299-shCtrl and H1299-shMAPK15 cells with/without TNF-α treatment (20 ng/mL, 24 h), crystal violet in the migrated cells (**C**) was dissolved and absorbance was measured at OD_595_ (**D**). (**E**) H1299 cells cultured in serum-reduced medium (1% FBS) were stimulated with 20 ng/mL TNF-α for 1 h and p50 localization was detected in H1299 cells; scale bar represents 80 μm. (**F**) H1299-shCtrl and H1299-shMAPK15 cells cultured in serum-reduced medium (1% FBS) were treated with/without 20 ng/mL TNF-α for 12 h and the expression of EP3 was detected. (**G**,**H**) H1299 cells transfected with control siRNA or EP3 siRNA were resuspended in serum-reduced medium (1% FBS) and seeded to transwell chamber with/without 20 ng/mL TNF-α. Crystal violet in the migrated cells (**G**) was dissolved and absorbance was measured at OD_595_ (**H**). NS, non-significant; * *p* < 0.05, Student’s *t*-test.

MAPK15 interacts with p50 intracellularly, indicating potential gene regulation and cellular phenotypic change. Beinke et al. reported that the p105 pathway can positively regulate gene transcription under TNF-α stimulation [23]. We hypothesize that TNF-α might promote the expression of EP3 through the p50 pathway, thereby contributing to cell migration. In TNF-α-treated H1299 cells, we found that TNF-α promoted EP3 expression in a dose- (Figure 4A) and time-dependent manner (Figure 4B). Furthermore, TNF-α promoted the migration of H1299 cells but had no significant effect on the migration of MAPK15 knockdown cells (Figure 4C,D). This result suggests that TNF-α promotes cell migration through MAPK15. In TNF-α-treated A549 cells, we found that TNF-α promotes nuclear localization of MAPK15 and p50 (Figure S2). In H1299 cells, we found that p50 is distributed in both cytoplasm and nucleus, whereas in MAPK15 knockdown cells, p50 is mainly located in the cytoplasm (Figure 4E), indicating that nuclear localization of p50 is dependent on MAPK15. At the same time, we treated H1299 cells with TNF-α and found that p50 is mainly located in the nucleus, whereas in MAPK15 knockdown cells, p50 is distributed in both the cytoplasm (white arrows) and the nucleus (Figure 4E). The above results indicate that TNF-α-induced nuclear translocation of p50 is dependent on MAPK15. In addition, we found that the expression of EP3 in TNF-α-treated H1299 cells was increased, while the expression changes of EP3 in MAPK15 knockdown H1299 cells were not significant (Figure 4F), and TNF-α could not promote H1299 cell migration while EP3 was knocked down (Figure 4G,H). Taken together, these results reveal that TNF-α promotes H1299 cell migration through induction of MAPK15-p50 nuclear localization and EP3 expression in cells with MAPK15 expression.

### 3.6. JSH-23 Inhibits MAPK15-Induced EP3 Expression and Cell Migration

**Figure 5:**
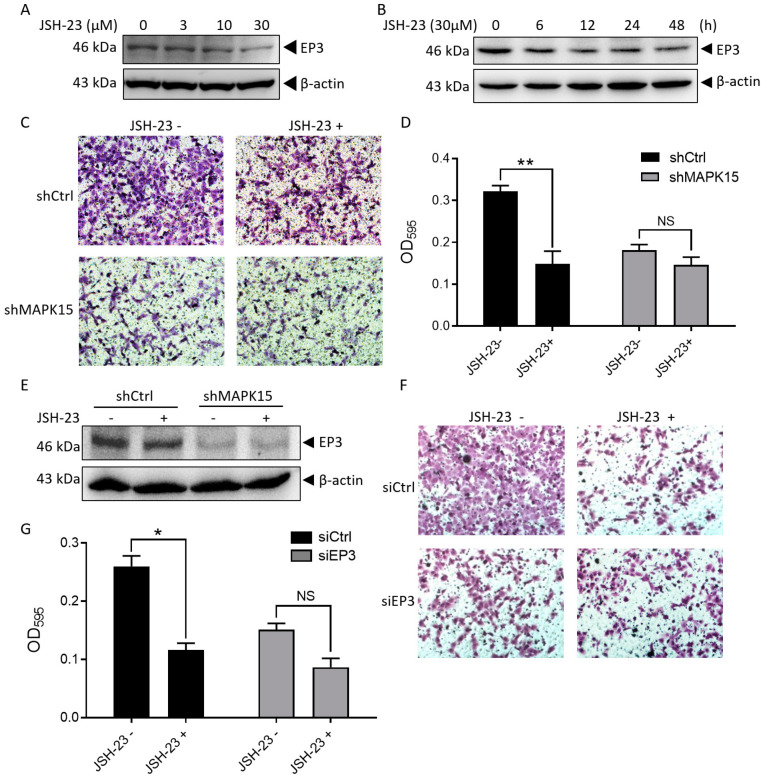
JSH-23 inhibits MAPK15-induced EP3 expression and cell migration. (**A**,**B**) H1299 cells were treated with/without different doses of JSH-23 for 12 h (**A**) or with 30 μM JSH-23 for different time points (**B**). The expression of EP3 was detected by immunoblot analysis. (**C**,**D**) H1299-shCtrl and H1299-shMAPK15 cells were resuspended in serum-reduced medium (1% FBS) and seeded to transwell chamber with/without 30 μM JSH-23, crystal violet in the migrated cells (**C**) was dissolved and absorbance was measured at OD_595_ (**D**). (**E**) H1299-shCtrl and H1299-shMAPK15 cells cultured in serum-reduced medium (1% FBS) were treated with/without 30 μM JSH-23 for 12 h and the expression of EP3 was detected. (**F**,**G**) H1299 cells transfected with control siRNA or EP3 siRNA were resuspended in serum-reduced medium (1% FBS) and seeded to transwell chamber with/without 30 μM JSH-23 for 24 h, crystal violet in the migrated cells (**F**) was dissolved and absorbance was measured at OD_595_ (**G**). NS, non-significant; * *p* < 0.05, ** *p* < 0.01, Student’s *t*-test.

JSH-23 is an NF-κB inhibitor. When using JSH-23 to treat H1299 cells, we found that JSH-23 inhibited the expression of EP3 in a dose- (Figure 5A) and time-dependent manner (Figure 5B). Furthermore, JSH-23 inhibited the migration of H1299 cells but had no significant effect on the migration of knockdown MAPK15 cells (Figure 5C,D). This result suggests that JSH-23 inhibits cell migration through MAPK15. In addition, we found that the expression of EP3 in H1299 cells treated with JSH-23 was decreased, while the expression of EP3 in MAPK15 knockdown H1299 cells did not change significantly (Figure 5E), and JSH-23 could not inhibit H1299 cell migration when EP3 was knocked down (Figure 5F,G). The above results indicate that JSH-23 inhibits cell migration by inhibiting MAPK15-induced EP3 expression.

## 4. Discussion

**Figure 6:**
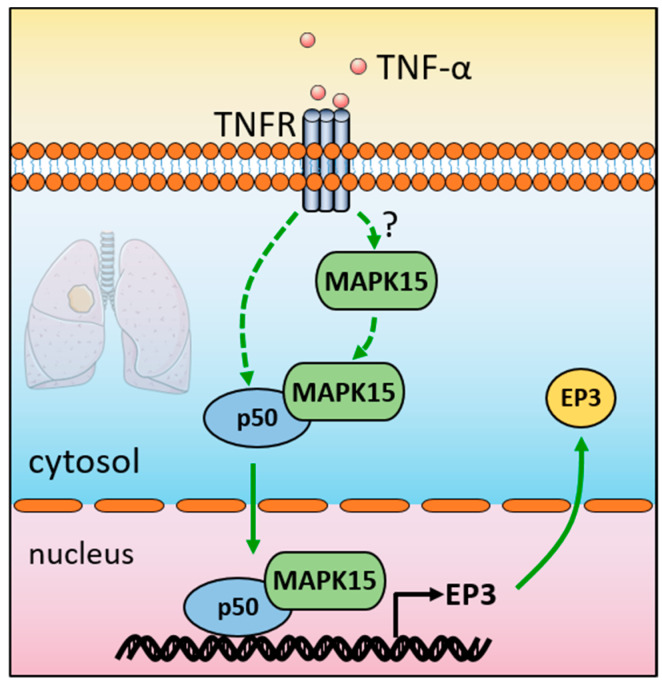
Schematic diagram of MAPK15 transcriptionally regulating EP3 by interacting with NF-κB p50 subunit and promoting LUAD metastasis. Question mark (?) indicates that how TNF-α affects MAPK15 in the cytosol is still unclear.

Lung cancer is usually at an advanced stage with lymph node or distant metastasis when diagnosed, which leads to high mortality. Medical knowledge still lacks effective diagnostic molecular markers for metastatic lung cancer. In the present study, we revealed that MAPK15 is more highly expressed in the tissues of LUAD patients with lymph node metastasis (Figure 1B), and MAPK15 interacts with p50 to promote EP3 expression at the transcriptional level (Figure 6), thereby enhancing cancer cell migration and metastasis.

MAPK15 is a member of the ERK subfamily, which is involved in the regulation of cell growth and differentiation like other well-known ERKs. Previous research indicates that MAPK15 is involved in the transformation of colon cancer [6], promotes gastric cancer cell proliferation [7], and is associated with autophagy [8]. However, its clinical pathological role has, until now, not been examined in lung cancer. The correlation between MAPK15 and lymph node metastasis in LUAD described here suggests that MAPK15 plays an important role in lung cancer development, which may lead to poor clinical outcomes. Since we used a commercialized lung cancer tissue array in this study, there is a lack of relevant information on disease progression, so it is impossible to conduct a longitudinal assessment of the relationship between MAPK15 expression and patients’ disease-free survival/overall survival, recurrence, metastasis, etc. However, with the in-depth study of MAPK15, we gradually realized its important role in LUAD. In future studies, multicenter, larger-sample-size studies should be conducted through longitudinal assessment of the patients’ critical long-term clinical outcome to further clarify MAPK15 expression and the significance of clinical parameters. Due to the significant correlation between MAPK15 and the clinical features of the LUAD patients we observed, MAPK15 and its signaling pathway in LUAD may be a potential therapeutic target for metastatic LUAD. As a kinase, MAPK15 carries out different functions in various cancers, indicating the deregulation of key pathways. Studies have indicated a pivotal role of MAPK15 in mediating the effect of gene transcription. We have previously shown that MAPK15 promotes the transformation of colon cancer by mediating the activation of c-Jun [6]. Here, the identification of MAPK15 as an upstream regulator for EP3 unveiled a previously unknown mechanism for the MAPK15 or EP3 signaling pathway and their roles in the regulation of cell migration in LUAD.

The role of EP3 in tumor progression is still controversial. It has been reported that EP3 coupled with G proteins can effectively inhibit tumor growth. Shoji et al. found that EP3 can significantly inhibit the proliferation of tumor cells in advanced-stage colon cancer [24]. Sanchez et al. found that EP3 can promote the expression of p21 by reducing cAMP, thereby arresting the cell cycle in the S phase, and ultimately inhibiting the proliferation of 3T6 fibroblasts [25]. On the other hand, there are more and more studies showing that EP3 can promote the development of tumors. Finetti et al. found that EP3 is involved in regulating the formation of tumor blood vessels [26]. Amano et al. found that in an EP3-deficient mouse tumor model, tumor angiogenesis and tumor cell growth were effectively inhibited [13]. Yamaki et al. found that EP3 participates in the Src signaling pathway to promote the growth of LUAD A549 cells [14]. In this study, we reveal that knocking down EP3 can inhibit the migration of LUAD cells and that the expression of EP3 was positively regulated by MAPK15, which expands our understanding of EP3 and its regulation in lung cancer.

NF-κB is a type of transcription factor that plays an important role in the occurrence and development of tumors. The ERK family was linked to the NF-κB pathway [27,28]. As the most recently discovered MAPK family member, the relation between MAPK15 and NF-κB is mainly uncharacterized. Previous studies on the NF-κB protein family mainly focused on the activity of IκB or p65 in the p50/p65 complex to promote gene transcription. However, more and more studies have shown that p50 can bind to the promoter of the gene and activate gene transcription. The study of Hong et al. showed that overexpression of p50 in BAR-T cells significantly enhanced the activity of the DNMT1 gene promoter [29]. Karst et al. showed that overexpression of NF-κB p50 in melanoma cells MMRU can promote angiogenesis and up-regulate IL6 expression. They confirmed by Chip assay that p50 can bind to the promoter region of IL6 gene and activate its transcription [30]. Similarly, Southern et al. found that the BAG-1 protein can interact with the p50-p50 homodimer and bind to the promoter region of downstream genes to play a positive role in regulating gene transcription [31]. Beinke et al. reviewed that TNF-α/IL-1/LPS can activate the classic p50/p65 dimer NF-κB signaling pathway and the p100/RelB non-canonical signaling pathway, as well as the p105/p50 signaling pathway [23]. In this study, we found that MAPK15 interacts with p50 in LUAD cells, and the nuclear translocation of p50 may require the assistance of MAPK15. In addition, we also found that the mRNA expression level of EP3 increased when p50 was overexpressed in H1299 cells, indicating that p50 can regulate the expression of EP3 at the transcriptional level, and CHIP assay and luciferase reporter assay confirmed that p50 can bind to the promoter region of EP3 and promote the transcription of EP3. Our results revealed that MAPK15 interacts with p50 to promote the transcription of EP3, thereby affecting biological functions such as the migration of LUAD cells.

## 5. Conclusions

In conclusion, this study demonstrates the role of MAPK15 in the metastasis of LUAD. We revealed that MAPK15 promotes LUAD cell migration via p50 and EP3 signaling and is associated with lymph node metastasis in LUAD patients, which indicates that MAPK15 might be a potential prognostic biomarker for LUAD and a therapeutic target to inhibit metastasis in metastatic LUAD patients. The insights provided by this study could facilitate understanding the role of MAPK15 in lung cancer progression and its potential modulatory role in cancer metastasis.

## Figures and Tables

**Table 1:** Correlation between MAPK15 expression and clinical parameters in patients with LUAD and LUSC.

	Adenocarcinoma				Squamous Cell Carcinoma			
Clinicopathological Parameters	MAPK15 Expression		Total	*p* Value	MAPK15 Expression		Total	*p* Value
	Low	High			Low	High		
Regional lymph nodes				0.013 *				0.486
N0	10	13	23		13	8	21	
N1	2	18	20		5	7	12	
N2	3	2	5		3	4	7	

Fisher’s exact test. Statistically significant, * *p* < 0.05.

**Table 2:** Correlation between EP3 expression and MAPK15 in LUAD tissues.

		EP3				Total
		−	+	++	+++	
**MAPK15**	**−**	10	5	0	0	15
	**+**	3	12	1	0	16
	**++**	0	5	1	1	7
	**+++**	2	2	3	3	10
**Total**		15	24	5	4	48

Spearman correlation, r = 0.589, *p* < 0.001.

## Data Availability

The data presented in this study are available on request from the corresponding author.

## Supplementary Materials

The following supporting information can be downloaded at: https://www.mdpi.com/article/10.3390/cancers15051398/s1, Table S1: Primer and siRNA used in this study; Table S2: Correlation between MAPK15 expression and clinicopathological parameters in patients with lung cancer; Table S3: Correlation between MAPK15 expression and tissue types; Figure S1: Matrix metalloproteinase-2 (MMP2) was significantly decreased in MAPK15 knockdown H1299 cells; Figure S2: Localization of MAPK15 and p50 in TNF-α treated A549 cells; Figure S3: Original blots and blot quantification of Figure 1; Figure S4: Original blots and blot quantification of Figure 2; Figure S5: Original blots and blot quantification of Figure 3; Figure S6: Original gels and gel quantification of Figure 3; Figure S7: Original blots and blot quantification of Figure 4; Figure S8: Original blots and blot quantification of Figure 5.
