# Supplementary material for: The Atypical MAP Kinase MAPK15 Is Required for Lung Adenocarcinoma Metastasis via Its Interaction with NF-κB p50 Subunit and Transcriptional Regulation of Prostaglandin E2 Receptor EP3 Subtype
Source: Cancers (Basel). 2023 Feb 22;15(5):1398. doi: 10.3390/cancers15051398 (PMC10000388; doi:10.3390/cancers15051398)
Supplement: Supplementary file 1 [file cancers-15-01398-s001.zip › cancers-2167681-supplementary.pdf]

Table S1 Primer and siRNA used in this study

Gene/siRNA	Sense (5′ to 3′)	Antisense (5′ to 3′)
β-actin	GAGCTACGAGCTGCCTGACG	CTCCATGCCCAGGAAGGAAGG
MAPK15	GACCAGAAGCCGTCCAATGT	GTATCGGTGCGAAGAGAGCA
EP3	CTCCGCTCCTGATAATGATGTTG	CTTTCTGCTTCTCCGTGTGTG
NF-κB p50	GCAGCTGGGAGATCGGGAAA	TTGTGAAGCTGCCAGTGCTA
MMP2	GTCCCCATGAAGCCCTGTTC	GCTGTCATAGGATGTGCCCT
EP3 siRNA	CGAACAGCUAUUAAGAAGAAGUUGC	GCAACUUCUUCUUAAUAGCUGUUCG

Table S2 Correlation between MAPK15 expression and clinicopathological parameters in patients with lung cancer

Clinicopathological parameters	MAPK15 expression		Total	*p* value
	Low	High		
Age (years)				0.688
<59	24	31	55	
≥59	22	33	55	
Gender				0.510
Male	36	46	82	
Female	10	18	28	
Depth of tumor invasion				0.100
T1+T2	40	47	87	
T3+T4	6	17	23	
Regional lymph nodes				0.012*
N0	31	27	58	
N1+N2	15	37	52	
Distant metastasis				1.000
M0	46	63	109	
M1	0	1	1	
Grade				0.621
1	2	1	3	
2	19	28	47	
3	13	22	35	
Stage				0.033*
I	29	26	55	
II+ III+ IV	17	38	55	

Fisher's exact test. Statistically significant, * $p<0.05$.

Table S3 Correlation between MAPK15 expression and tissue types

	MAPK15 expression		Total	χ2	*p* value
	Low	High			
Histological type				4.076	0.044*
Adenocarcinoma	15	33	48		
Squamous cell lung carcinoma	21	19	40		

Chi-square test (χ2). Statistically significant, * $p<0.05$.

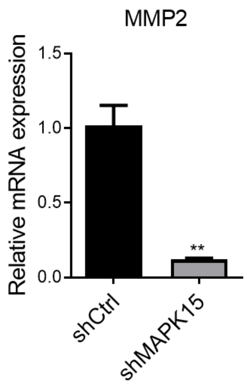

Figure S1. Matrix metalloproteinase-2 (MMP2) was significantly decreased in MAPK15 knockdown H1299 cells. ** $p < 0.01$, Student's t-test.

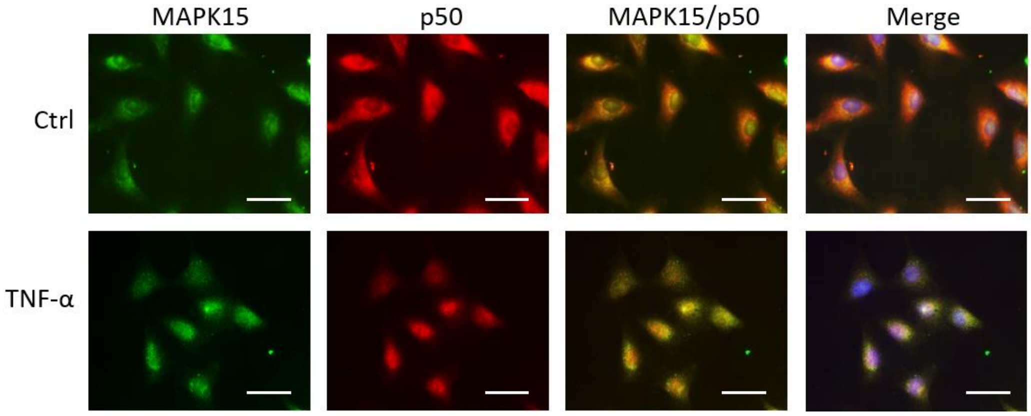

Figure S2. Localization of MAPK15 and p50 in TNF-α treated A549 cells. A549 cells were treated with/without TNF-α and antibodies against MAPK15 and p50 were used to detect the localization of MAPK15 and p50.

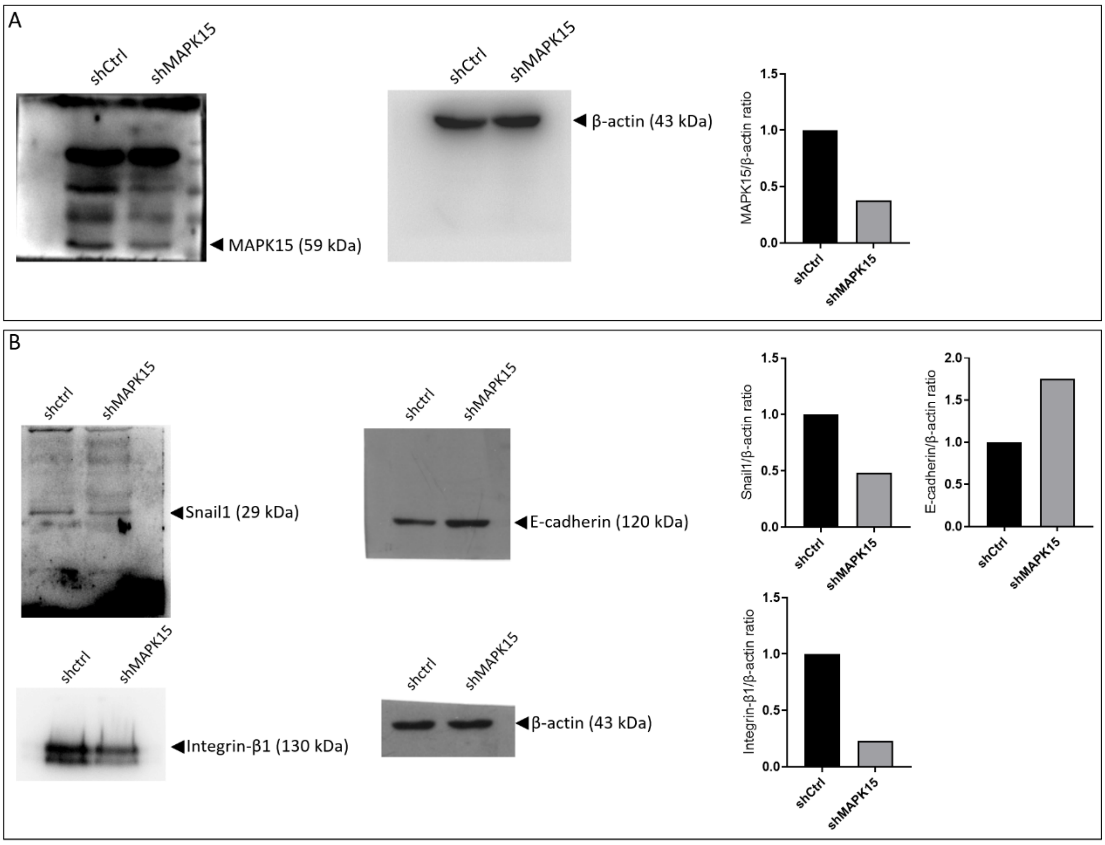

Figure S3. Original blots and blot quantification of Figure 1. Immunoblot analysis of MAPK15, Snail1, E-canherin and Integrin-β1 that correspond to Figure 1D (A) and Figure 1G (B).

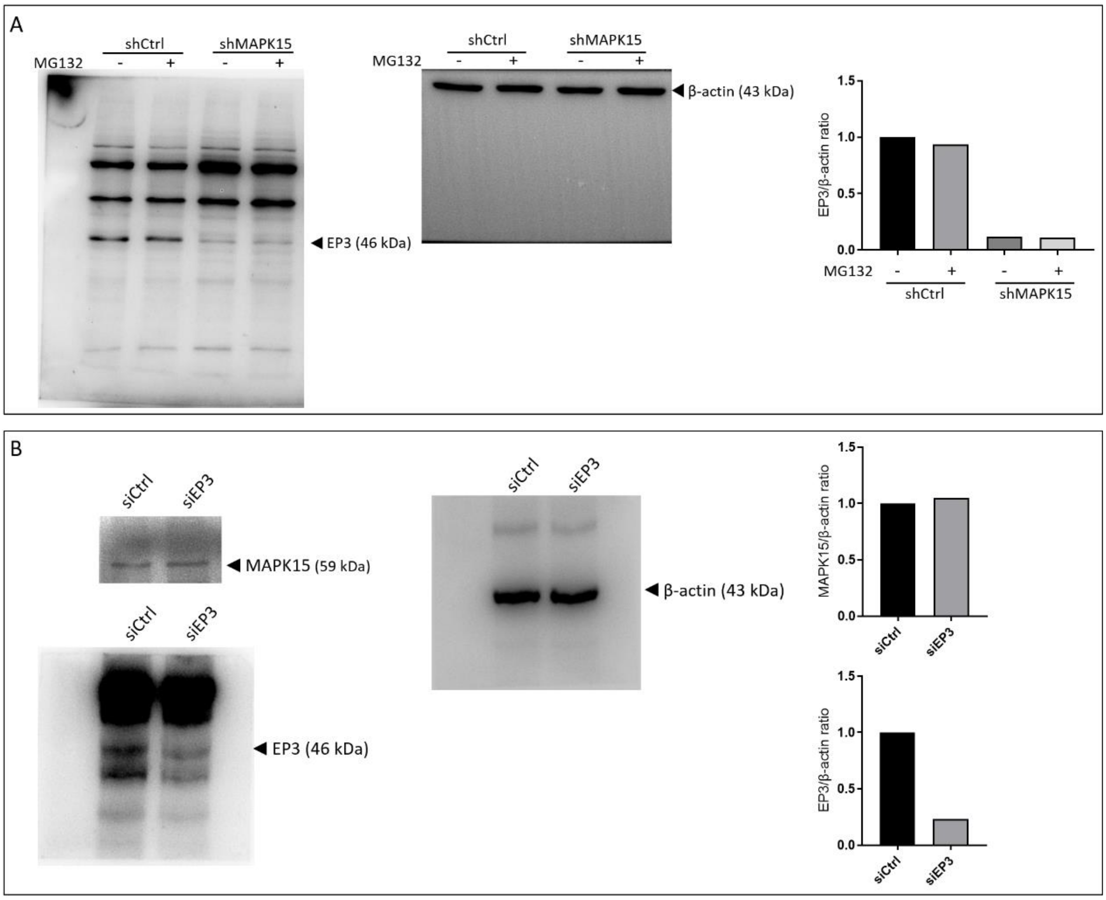

Figure S4. Original blots and blot quantification of Figure 2. Immunoblot analysis of EP3 and MAPK15 that correspond to Figure 2B (A) and Figure 2E (B).

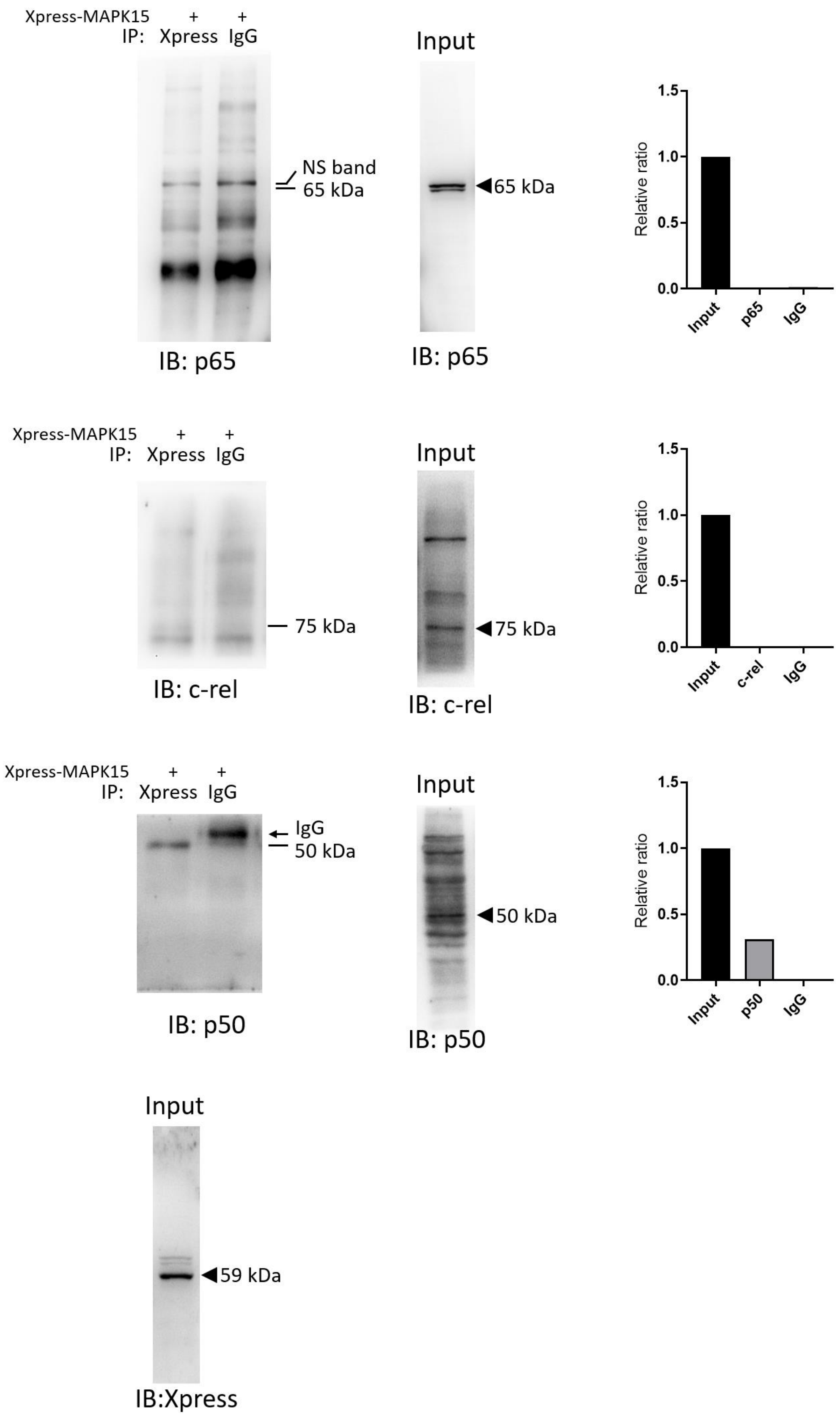

Figure S5. Original blots and blot quantification of Figure 3. Immunoblot analysis of co-immunoprecipitation that corresponds to Figure 3A. NS, non-specific.

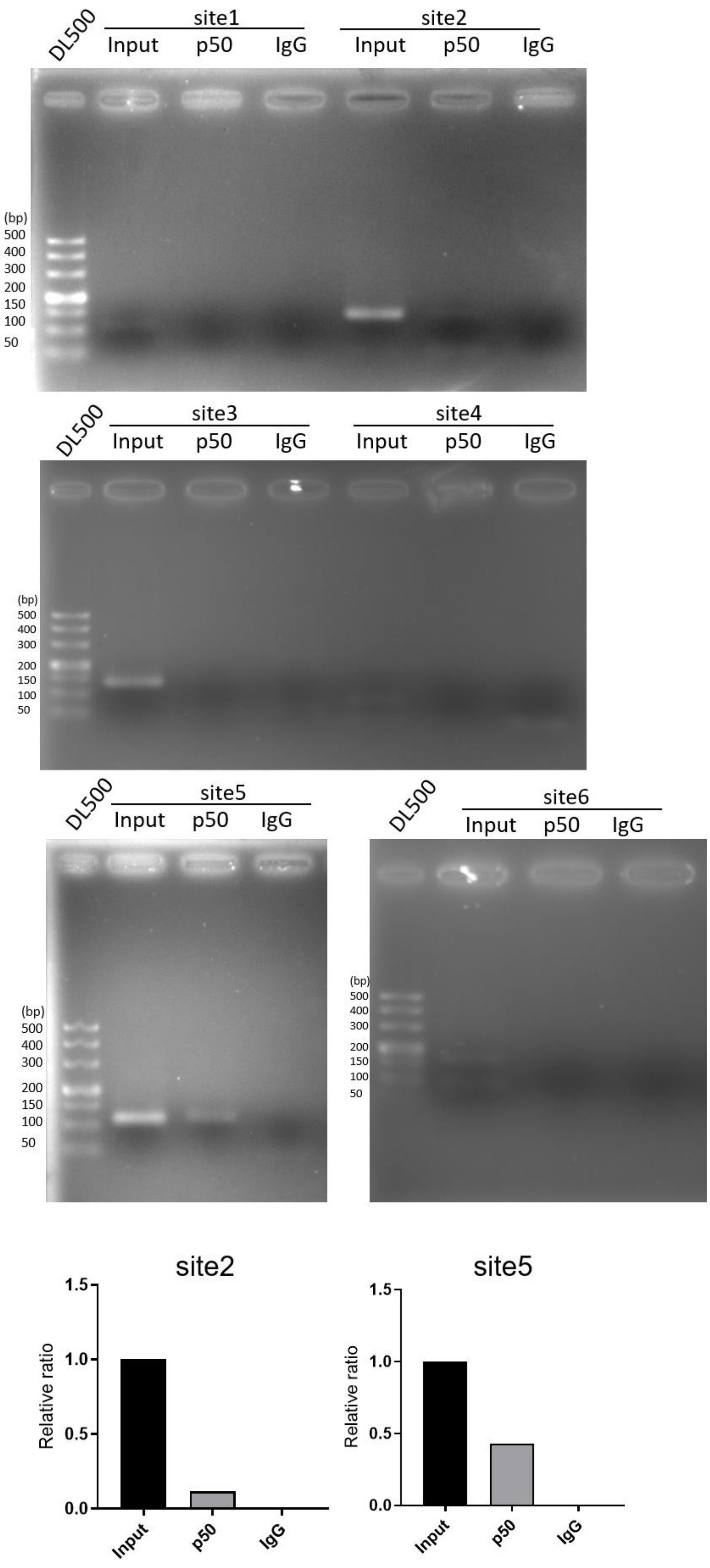

Figure S6. Original gels and gel quantification of Figure 3. Agarose gel electrophoresis results that correspond to Figure 3F.

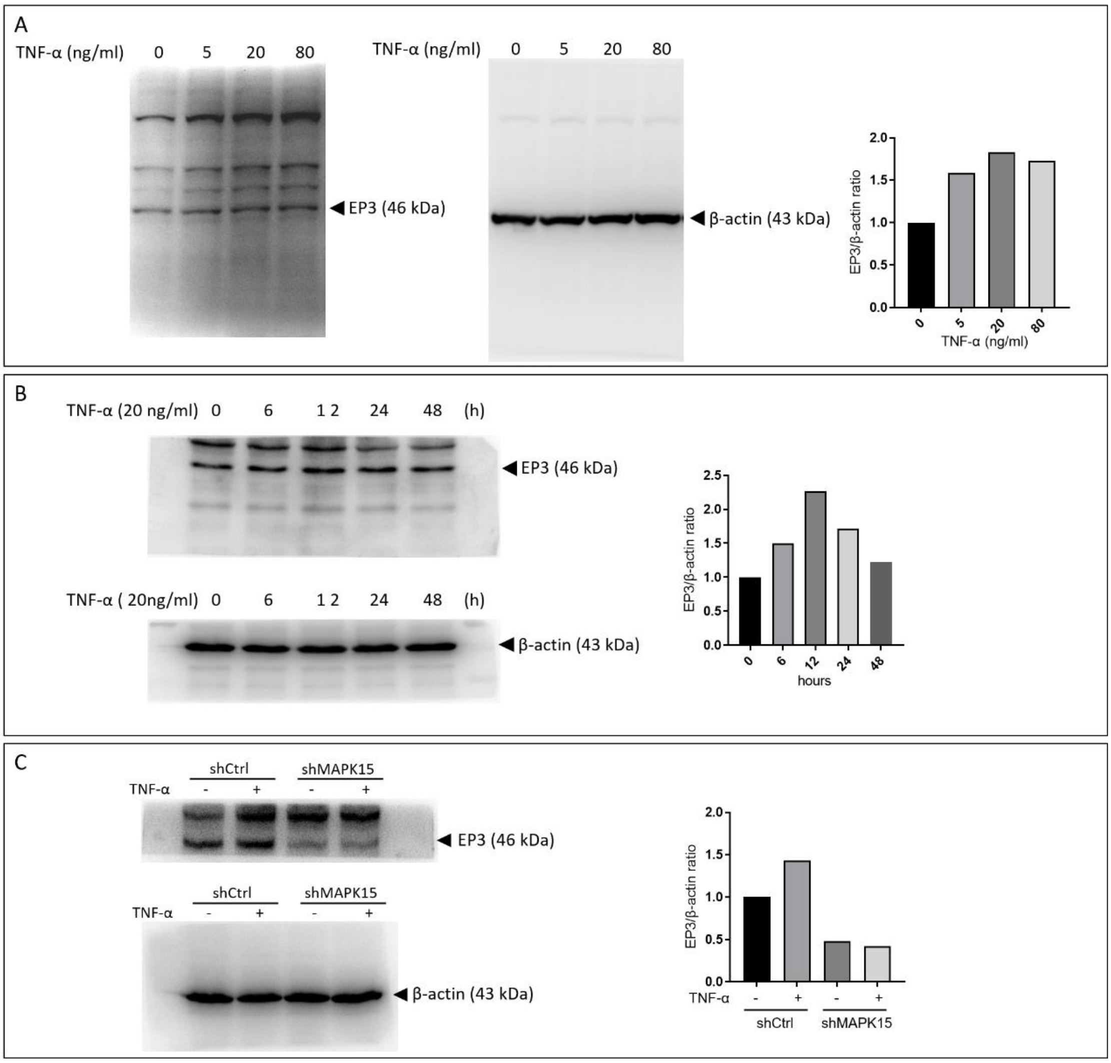

Figure S7. Original blots and blot quantification of Figure 4. Immunoblot analysis of EP3 expression corresponds to Figure 4A (A), Figure 4B (B), Figure 4F(C).

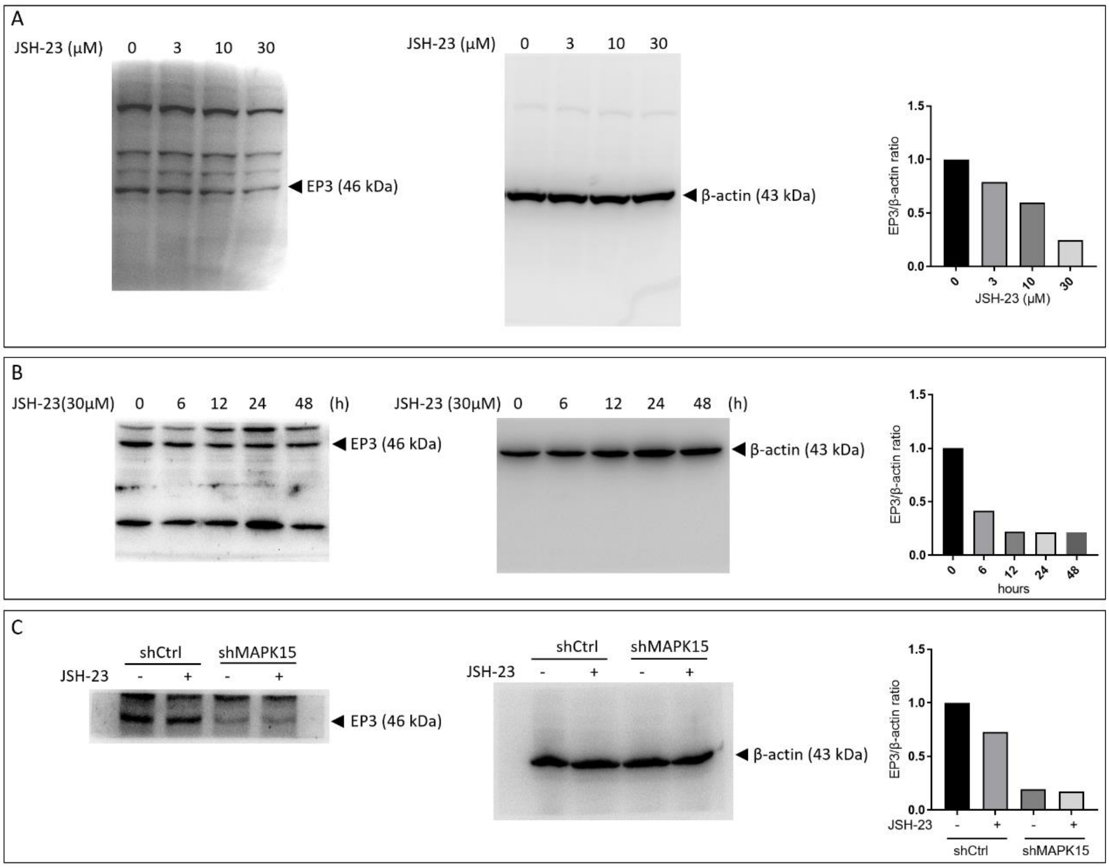

Figure S8. Original blots and blot quantification of Figure 5. Immunoblot analysis of EP3 expression corresponds to Figure 5A (A), Figure 5B (B), and Figure 5E(C).
